# Supplementary material for: Genome-wide identification and expression analysis of new cytokinin metabolic genes in bread wheat (Triticum aestivum L.)
Source: PeerJ. 2019 Jan 31;7:e6300. doi: 10.7717/peerj.6300 (PMC6360083; doi:10.7717/peerj.6300)
Supplement: Table S1 [file peerj-07-6300-s001.pdf]

Table S1. Primers used in this study.

Forward Primer	Sequence	Reverse Primer	Sequence
TaGLU1d-F	GACCAAGGCGCAGACGTA	TaGLU1d-R	GGGAGCTTGCCCAGTTGG
TaTaGLU2-F	GGTTAAACTACTACACCTCACGG	TaGLU2-R	GCCTCCATGGGTAGAGGTG
TaGLU3-F	CCATTGTCGGATGTCGATGAA	TaGLU3-R	CCTTACCCGCATCCTGTCG
TaGLU4-F	GACCCAGGGGCATGGAAC	TaGLU4-R	CGTGAACACCGTCAGCGAT
TaGLU5-F	CGGAGAAAAATATCGAGCTGCC	TaGLU5-R	GCTCTAGTATCCATCCGAACAC
TaGLU6-F	CACCTACTACACCAGGCACA	TaGLU6-R	GGCGTCCTTGAGGGCAA
TaGLU7-F	CGGGAGCAGTACTTGCGG	TaGLU7-R	CAGTGCTGCAGAAGGTCTCT
TaGLU9-F	ACCCGCTTACAAATTCCACAG	TaGLU9-R	GTCCGTGTTGTAGTCCCGA
TaGLU12-F	GCTTGAGGAACAGCTACTCTACT	TaGLU12-R	GCACTCAGCAGGGCATCA
TaGLU13-F	CGTCAGGTATGTCAATCAGCG	TaGLU13-R	CTTTGGAGTCCTCTCTTGTGTTTC
TaGLU30-F	GCCGACGAGACTGCCTTC	TaGLU30-R	GATTGCAGGGTTTCCATACTTGAG
TaCKX1-F	GTGGCGCTGGACAAGATC	TaCKX1-R	AGAACCGCAGTATCTTCTGGT
TaCKX2-F	GCTCTATACTACGGCGGCG	TaCKX2-R	GCGGGGGAGGAAGAGGT
TaCKX3-F	CCTCTCTGCCTCGTCTCTAG	TaCKX3-R	CCAGCCTTCAAGCTCTGC
TaCKX4-F	CGGCTGAGATACATACAGTCAAC	TaCKX4-R	CGTCCTGTTGTCCCACTTTG
TaCKX5-F	ATTGCCGGTGTACTCGCC	TaCKX5-R	CTCCGGGTTCTTCGCCTC
TaCXK7-F	TTCTCGACTTTGACGCTGGG	TaCKX7-R	TGTAGAATACGTCCTCGCCAG
TaCKX8-F	AACGTCCTGCAGCTCCAA	TaCKX8-R	CTGCGGATGGAGTGCTCAT
TaCKX9-F	CATTCAATCCACAGGACCCAGAA	TaCKX9-R	CTGAGTTGAGATAGTAGTGCATGG
TaCKX10-F	TGAACCGGACCCTGACG	TaCKX10-R	CTTCTGCTCCTCCTGCCC
TaCKX11-F	GCCCTGTACCAGCACCAC	TaCKX11-R	ACAGGAACTCCACGTACCC
TaCisZOG1-F	GAGAAGGAGATGGAGGAGCG	TaCisZOG1-R	GCTGCTTGTCGAGCCAG
TaCisZOG3-F	GGAGAAGGAGATGGAGGAACA	TaCisZOG3-R	GACACGTAGAGCACCGATGA
TaZOG3-F	CGCAGGTGACCATCCTCT	TaZOG3-R	CTCCTCGGGGACGTTCTT
TaZOG2-F	ACCGAGCAGCTGGTCTCA	TaZOG2-R	TTCACCAGGTCGTTCCTCA
TaZOG4-F	GAGGGGCTGGAGAACGC	TaZOG4-R	GCACCTCGAACTCCTCCATG
TaIPT1-F	GGATAAGATCCAGGTGTACGCT	TaIPT1-R	GAGGCAGTCATGGGAGGAG
TaIPT2-F	AACACGTTCGGCCTCGT	TaIPT2-R	CAGATCAGTTGGCAGGGTAGAA
TaIPT3-F	CTTCCTCGCCAAGCGATTT	TaIPT3-R	TCAGAGGCCGACGTTGTC
TaIPT6-F	CGCACCGACTACTCTCGT	TaIPT6-R	CCGCTCTTGAGGAACACCT
TaIPT7-F	CGAGCGGCAATGGCAAAG	TaIPT7-R	GTGGCCTTGTTGGTGACC
TaIPT8-F	CTCTGGGTGGACGTGGAG	TaIPT8-R	TGCGCCGGATCTTGGAC
TaIPT9-F	TTGCCGCGATTTCCGTGA	TaIPT9-R	ACCCACTTCCAACACCATCT
TaIPT10-F	GTGGGAAGAAGCGGTGGAG	TaIPT10-R	GCATCATCGCTTGGGGAATC
