# Supplementary material for: Genome-wide identification and expression analysis of new cytokinin metabolic genes in bread wheat (Triticum aestivum L.)
Source: PeerJ. 2019 Jan 31;7:e6300. doi: 10.7717/peerj.6300 (PMC6360083; doi:10.7717/peerj.6300)
Supplement: Table S2 [file peerj-07-6300-s002.pdf]

Gene IDs and genomic sequences of TaCKX gene family

NAME	GENE ID
TaCKX1AA	TraesCS3A01G109500
TaCKX1BB	TraesCS3B01G128700
TaCKX1DD	TraesCS3D01G111300
TaCKX2AA	TraesCS3A01G311000
TaCKX2BB	TraesCS3B01G161100
TaCKX2DD	TraesCS3D01G143600
TaCKX3AA	TraesCS1A01G159600
TaCKX3BB	TraesCS1B01G176000
TaCKX3DD	TraesCS1D01G157000
TaCKX4AA	TraesCS3A01G481000
TaCKX4BB	TraesCS3B01G525300
TaCKX4DD	TraesCS3D01G475800
TaCKX5AA	TraesCS3A01G321100
TaCKX5BB	TraesCS3B01G344600
TaCKX5DD	TraesCS3D01G310200
TaCKX7AA	TraesCS6A01G185800
TaCKX7BB	TraesCS6B01G214700
TaCKX7DD	TraesCS6B01G214700
TaCKX8AA	TraesCS2A01G378300
TaCKX8BB	TraesCS2B01G395200
TaCKX8DD	TraesCS2D01G374600
TaCKX9AA	TraesCS1A01G234800
TaCKX9BB	TraesCS1B01G248700
TaCKX9DD	TraesCS1D01G237200
TaCKX10AA	TraesCS7A01G363400
TaCKX10BB	TraesCS7B01G264400
TaCKX10DD	TraesCS7D01G359700
TaCKX11AA	TraesCS7A01G536900
TaCKX11BB	TraesCS7B01G455000
TaCKX11DD	TraesCSU01G106300
TaCKX12DD	TraesCS3D01G143500
TaCKX13DD	TraesCS3D01G143200
TaCKX14AA	TraesCS3A01G311100
TaCKX14BB	TraesCS3B01G161000
TaCKX14DD	TraesCS3D01G143300

Gene IDs and genomic sequences of TaZOG gene family

NAME	GENE ID
TaZOG1AA	TraesCS5A01G444500
TaZOG1BB	TraesCS5B01G528900
TaZOG1DD	TraesCS5D01G527900
TaZOG2AA	TraesCS5A01G553100
TaZOG2BB	TraesCS4B01G388700
TaZOG2DD	TraesCSU01G124500
TaZOG3AA	TraesCS3A01G125500
TaZOG3BB	TraesCS3B01G144600
TaZOG4AA	TraesCS7A01G189500
TaZOG4BB	TraesCS7B01G094400

TaZOG4DD	TraesCS7D01G190600
TacisZOG1AA	TraesCS2A01G410000
TaCisZOG1BB	TraesCS2B01G428300
TaCisZOG1DD	TraesCS2D01G407200
TacisZOG3AA	TraesCS2A01G474700
TaCisZOG3BB	TraesCS2B01G498300
TaCisZOG3DD	TraesCS2D01G475100
TacisZOG4AA	TraesCS7A01G467800
TaCisZOG4BB	TraesCS7B01G369200
TaCisZOG4DD	TraesCS7D01G455200

Gene IDs and genomic sequences of TaIPT gene family

NAME	GENE ID
TaIPT1AA	TraesCS5A01G181100
TaIPT1BB	TraesCS5B01G178800
TaIPT1DD	TraesCS5D01G185500
TaIPT2AA	TraesCS1A01G057000
TaIPT2BB	TraesCS1B01G074400
TaIPT2DD	TraesCS1D01G067500
TaIPT3AA	TraesCS3A01G263300
TaIPT3BB	TraesCS3B01G296500
TaIPT3DD	TraesCS3D01G263000
TaIPT4BB	TraesCS1B01G323900
TaIPT6AA	TraesCS5A01G460000
TaIPT6BB	TraesCS5B01G469600
TaIPT6DD	TraesCS5D01G471100
TaIPT7AA	TraesCS3A01G136200
TaIPT7BB	TraesCS3B01G154100
TaIPT7DD	TraesCS3D01G137000
TaIPT8AA	TraesCS1A01G376300
TaIPT8BB	TraesCS1B01G396700
TaIPT8DD	TraesCS1D01G383600
TaIPT9AA	TraesCS2A01G265700
TaIPT9BB	TraesCS2B01G276300
TaIPT9DD	TraesCS2D01G258000
TaIPT10AA	TraesCS7A01G560300
TaIPT10BB	TraesCS7B01G486700
TaIPT10DD	TraesCS7D01G549300

Gene IDs and genomic sequences of newly identified TaGLU genes

NAME	GENE ID
TaGLU5AA	TraesCS3A01G447500
TaGLU5BB	TraesCS3B01G483500
TaGLU5DD	TraesCS3D01G440200
TaGLU6AA	TraesCS4A01G056400
TaGLU6BB	TraesCS4B01G248600
TaGLU6DD	TraesCS4D01G248000
TaGLU7AA	TraesCS4A01G272900
TaGLU7BB	TraesCS4B01G041100
TaGLU7DD	TraesCS4D01G038500
TaGLU8AA	TraesCS4A01G272800
TaGLU8BB	TraesCS4B01G041200
TaGLU8DD	TraesCS4D01G038600

TaGLU9BB	TraesCS3B01G368800
TaGLU9DD	TraesCS3D01G418000
TaGLU11AA	TraesCS3A01G448200
TaGLU11BB	TraesCS3B01G484200
TaGLU11DD	TraesCS3D01G440600
TaGLU12AA	TraesCS2A01G521000
TaGLU12BB	TraesCS2B01G820000
TaGLU12DD	TraesCS2D01G522700
TaGLU13AA	TraesCS2A01G384300
TaGLU13BB	TraesCS2B01G401500
TaGLU13DD	TraesCS2D01G381000
TaGLU14AA	TraesCS2A01G384600
TaGLU14DD	TraesCS2D01G381300
TaGLU15AA	TraesCS2A01G384400
TaGLU15DD	TraesCS2D01G381100
TaGLU16AA	TraesCS2A01G038400
TaGLU16DD	TraesCS2D01G037400
TaGLU17BB	TraesCS3B01G277600
TaGLU19AA	TraesCS3A01G447800
TaGLU19BB	TraesCS3B01G484000
TaGLU19DD	TraesCS3D01G440500
TaGLU21DD	TraesCS5D01G404700
TaGLU22BB	TraesCS3B01G369200
TaGLU22DD	TraesCS3D01G331000
TaGLU23AA	TraesCS1A01G229300
TaGLU23BB	TraesCS1B01G244800
TaGLU23DD	TraesCS1D01G232700
TaGLU24AA	TraesCS7A01G227300
TAGLU24BB	TraesCS7B01G192200
TaGLU24DD	TraesCS7D01G227100
TaGLU26AA	TraesCS3A01G003900
TaGLU26BB	TraesCS3B01G006000
TaGLU26DD	TraesCS3D01G006900
TaGLU28AA	TraesCS7A01G246300
TaGLU28BB	TraesCS7B01G145100
TaGLU28DD	TraesCS7D01G245000
TaGLU30AA	TraesCS5A01G265800
TaGLU30BB	TraesCS5B01G265500
TaGLU30DD	TraesCS5D01G273700
TaGLU31AA	TraesCS3A01G216000
TaGLU31BB	TraesCS3B01G195600
TaGLU31DD	TraesCS3D01G171000
TaGLU32AA	TraesCS5A01G295100
TaGLU32BB	TraesCS5B01G294400
TaGLU32DD	TraesCS5D01G302600
TaGLU34BB	TraesCS7B01G267800
TaGLU34DD	TraesCS7D01G363100
TaGLU35AA	TraesCS3A01G175800
TaGLU35BB	TraesCS3B01G205500
TaGLU35DD	TraesCS3D01G181400
TaGLU38AA	TraesCS7A01G386200
TaGLU38BB	TraesCS7B01G289100
TaGLU38DD	TraesCS4D01G085600
